# Selecting differential splicing methods: Practical considerations for short-read RNA sequencing

**DOI:** 10.12688/f1000research.155223.2

**Published:** 2025-05-30

**Authors:** Ben J. Draper, Mark J. Dunning, David C. James

**Affiliations:** 1 Department of Chemical and Biological Engineering, Mappin St., The University of Sheffield, Sheffield, S1 3JD, UK; 2 Bioinformatics Core Bioinformatics Core, The Faculty of Health,, The University of Sheffield, Sheffield, S10 2HQ, UK

**Keywords:** Bioinformatics, Alternative Splicing, RNASeq, Transcriptomics, Differential Expression

## Abstract

Alternative splicing is crucial in gene regulation, with significant implications in clinical settings and biotechnology. This review article compiles bioinformatics short-read RNA-seq tools for investigating differential splicing; offering a detailed examination of their statistical methods, case applications, and benefits. A total of 22 tools are categorised by their statistical family (parametric, non-parametric, and probabilistic) and level of analysis (transcript, exon, and event). The central challenges in quantifying alternative splicing include correct splice site identification and accurate isoform deconvolution of transcripts. Benchmarking studies show no consensus on tool performance, revealing considerable variability across different scenarios. Tools with high citation frequency and continued developer maintenance, such as DEXSeq and rMATS, are recommended for prospective researchers. To aid in tool selection, a guide schematic is proposed based on variations in data input and the required level of analysis. Emerging long-read RNA sequencing technologies are discussed as a complement to short-read methods, promising reduced deconvolution needs and further innovation.

## Introduction

Alternative splicing (AS) can be best described as fine-tuning gene expression by rearranging exons and introns in pre-mRNA. With 90-95% of human multi-exon genes estimated to possess some form of AS, it is a widespread regulatory process in cellular biology. ^1^ The cell utilises a large ribonucleoprotein (RBP) complex known as the spliceosome which is guided to target sites through the interaction of sequence elements (splice sites, enhancers & silencers and the polypyrimidine tract) and/or splicing factors. Pre-mRNA splicing can also occur without the splicesome as in the case of self-splicing group I & II introns, tRNA splicing and trans-splicing. ^2^ This ultimately results in genome-wide transcript diversity and subsequently, measurable changes to protein functionality.

Previous research has uncovered the phenotypic consequences of AS in disease. In humans, clinical research has shown AS as a key instigator in several forms of cancer and neurodegenerative disorders. ^3– 5^ One notable discovery in Microtubule-associated protein tau’s (MAPT) possession of mis-spliced isoforms causing abnormal TAU accumulation progressing to Alzheimer’s disease. ^6^ In cancer, numerous mis-spliced variants of tumour suppressors, apoptotic and angiogenic proteins have been discovered to contribute to tumour progression. ^7, 8^ Within the context of aging, the “energy-splicing axis hypothesis” further underscores AS’s prominent role in controlling phenotype. ^9^ Beyond clinical research, the utility of alternative transcripts for bioengineering purposes has been explored. For example, an alternatively spliced version of the transcription factor X-box binding protein 1 (XBP1) coexpressed in production cell lines has been shown to increase productivity in the biomanufacturing of recombinant proteins. ^10– 12^ In bio-agriculture, the CRISPR-mediated directed evolution of SF3B1 mutants (a spliceosomal component) in rice has improved crop traits through better resistance to splicing inhibitors. ^13^ Increasingly, the value of AS in both clinical and biotechnology applications has been recognised; highlighting the need for robust bioinformatics pipelines to identify variants.

For prospective researchers investigating AS, the transcriptomic data is typically generated using next-generation sequencing. Short-read RNAseq is the most commonly used experimental technique to interrogate a transcriptome owing to its versatility and cost-effectiveness. ^14, 15^ It involves sequencing short fragments of RNA molecules, providing insights into the respective expression levels of genomic features assembled from reference genomes. These features may be coding sequences, genes, transcripts, exons, introns, codons or even untranslated regions. Notably, the term “gene” refers to the DNA template, while “transcript” denotes the RNA molecules transcribed from it, as per recent nomenclature guidelines. ^16^

A typical RNAseq pre-processing pipeline will consist of quality control (QC), read alignment & quantification before statistical analysis begins. QC assesses the quality of the raw fragmented reads using a standardised tool such as FastQC and trims low-quality reads or adaptor sequences. ^17^ Then for alignment, a reference genome/transcriptome arranges the subsequent sequences into feature bins such as genes, transcripts, exons and coding sequences using software such as STAR or HISAT. ^18, 19^ Alignment files (usually in the form of Sequence Alignment Maps: SAMs) can then be quantified to these features using a quantification tool such as HTSeq, Salmon or featureCounts usually normalising for library size and sequencing depth. ^20– 22^ Depending on the purpose of analysis, normalisation may be scaled by total number of reads (CPM: Counts per Million), per length of transcript (TPM: Transcripts per Million), by paired-end fragments (RPKM: Fragments Per Kilobase of Transcript) or by using a median of ratios (DESeq2’s method). ^23^ Commonly, a differential expression analysis will be performed at the gene or transcript level between groups of samples to identify statistically significant changes in expression. While gene-level analysis aggregates all transcripts aligned to a gene, transcript-level analysis enables the study of specific isoforms, which is particularly relevant for AS-focused pipelines.

The pre-processing steps for RNA-seq have been extensively researched over many years, and there is a consensus within the community regarding the gold-standard set of tools. Projects like nf-core enable the execution of RNA-seq pre-processing pipelines with minimal intervention and limited bioinformatics expertise. ^24^ However, these tend to be focused on the use-case of conventional differential expression rather than the more bespoke AS pipelines as discussed here.

A growing repertoire of tools now annotate and quantify changes to splicing events. Quantification of features such as splice sites, and exon/intron junctions found in alignment files are commonly used to annotate splicing events. Although the true repertoire of splicing events is difficult to capture, conventional processes can be categorised into distinct groups. The most common events are exon skipping, retained introns, mutually exclusive exons, alternative 5′ and 3′ splice sites. Additional regulatory events involve genomic features like alternative transcription start sites (TSS) and polyadenylation sites, which lead to variations in mRNA 5′ and 3′ untranslated regions (UTRs), themselves exons. These events are less frequently analysed in standard bioinformatics pipelines, not due to greater biological complexity, but because short-read sequencing with typical library preparation methods (e.g., random hexamer priming) often lacks sufficient coverage of transcript 5′ and 3′ ends. ^25^ Specialized library preparations, such as Cap Analysis of Gene Expression (CAGE) for 5′ end capture or 3′ end sequencing (e.g., QuantSeq) for polyadenylation analysis, are required for these studies, with tools like CAGER and DaPars (Dynamic Analysis of Alternative Polyadenylation from RNA-Seq) supporting such niche research. ^26, 27^ Visualisation of AS is predicated upon the level of detail required in the analysis. If a highly detailed analysis of individual gene structure is needed, splice graphs, sashimi plots and junction maps are commonly used. ^28, 29^ To visualize changes to groups of transcripts, typically MA and Volcano plots are used much the same way as in differential expression level analysis. ^23^

## Current statistical methods for differential splicing

Commonly, researchers are interested in comparisons of two or more groups of samples known as differential analyses. Differential gene/transcript expression (DGE/DTE) of genes or transcripts involves taking raw read count data, normalizing or scaling it, and calculating whether the changes in expression levels between different biological groups are statistically significant. Differential transcript/exon usage (DTU/DEU), however, uses gene-level group modelling to assess whether the proportional use of the feature (exon or transcript) is statistically significant. Differential splicing events (DSE) on the other hand use a diverse array of statistical methods to quantify and infer splicing events. A comprehensive summary of differential splicing tools is described in the supplementary table ( **Supplementary Table 1**) and in the following sections.

### Parametric & mixed methods

**Figure 1.**
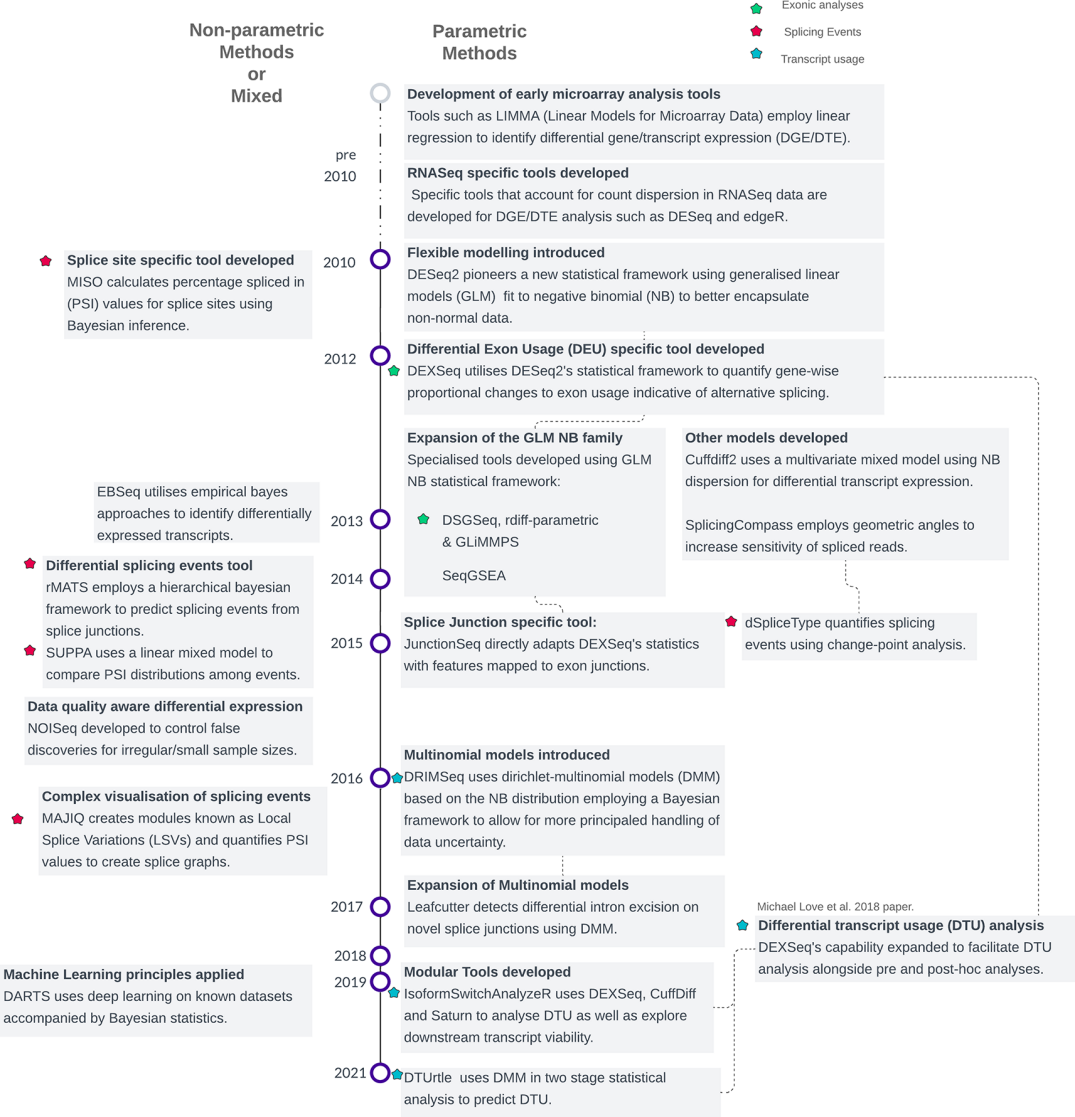
Timeline of statistical methods in differential splicing tool development. Methods are categorized into parametric and non-parametric approaches, grouped by methodological families. The classification is based on the underlying statistical procedures used for modelling or hypothesis testing, as detailed in Supplementary Table 1. Note that some methods incorporate elements of both parametric and non-parametric frameworks, resulting in overlapping features.

Differential expression analysis tools began in the early 2000s coinciding with the development of high throughput technologies such as microarrays. An early example was LIMMA (Linear Models for Microarray Data), developed by Gordon Smyth and colleagues in 2003, which utilises a linear regression framework and empirical Bayes techniques to identify differentially expressed features. ^30^ Whilst initially only utilised for microarrays, the functionality thus extended to RNASeq data and has been one of the most cited RNASeq methods. As the field shifted from microarray technology to RNASeq, methods were developed such as DESeq (Differential Expression Analysis for Sequence Count Data) and edgeR to capture the nature of count data better and improve modelling. ^23, 30, 31^ A major change incorporated in DESeq2 was empirical Bayes-based shrinkage to improve gene-wise variance estimation enhancing accuracy ( Figure 1). Secondly, GLMs (Generalized Linear Models) replaced the simple linear models as these were shown to adapt well to non-normally distributed count-based data. ^23^ The flexibility of GLMs allowed algorithms to effectively deal with issues such as overdispersion, shrinkage, heteroscedasticity and covariates. To date, GLMs are usually fitted to the NB (Negative Binomial) distribution which confers some strong advantages. The NB distribution effectively captures overdispersion (the empirical variability of counts) and can handle a large excess of zero values commonly seen in transcript or exon-level count data. However, limma, DESeq2 and edgeR were not developed to specifically address the challenges of identifying AS.

In 2014, DEXSeq was introduced by Michael Love and colleagues, a framework based on DESeq2’s GLM NB model becoming the de-facto tool for parametric splicing-based analysis. Instead of analysing gene-level differential expression, DEXSeq identifies exons within genes that exhibit significant changes in their usage across conditions. This is particularly useful for studying the exonic composition of alternatively spliced transcripts. The development of tools such as DSGseq, rDiff-parametric, JunctionSeq and SeqGSEA has expanded the functionality of the GLM NB family of differential splicing tools. ^32– 35^ DSGseq utilises a holistic approach considering splicing events not as individual elements but as comprehensive gene-wise splice graphs that more accurately reflect complex splicing dependencies. ^32^ The tool rDiff-parametric on the other hand utilises isoform-specific loci such as restricted exonic regions to identify significant differences in isoform composition. ^33^ By focusing on exonic regions unique to specific isoforms, rDiff-parametric avoids assigning ambiguous reads to overlapping isoforms. Assigning reads to isoforms is challenging because these transcripts are practically identical, making it difficult to definitively attribute a read from an overlapping region to a particular region without supplementary data. Therefore, full isoform deconvolution is significantly biased against genes with many isoform variants. ^36^

A few newer methods such as DRIMSeq and DTUrtle use non-parametric or mixed Dirichlet Multinomial Models (DMM) which have been argued to capture better the complex variability of count data and better estimate isoform abundance ^37, 38^ ( Figure 1 & **Supplementary Table 1**). Other methods such as IsoformSwitchAnalyzeR and some custom DEXSeq workflows now incorporate modularity allowing users a selection of bioinformatics tools for filtering, hypothesis testing and posterior calculations. ^39, 40^ An example of the usage of parametric analysis was in the discovery of a chimeric fusion transcript of PRKACA and DNAJB1 in a rare liver tumour FL-HCC (fibrolamellar hepatocellular carcinoma) using DEXSeq’s differential exon usage framework. ^41^ The discovery of differential exon usage of PRKACA’s exons 2-10 and subsequent decreased usage of DNAJB1’s exons 2-3 led the researchers to identify a chimeric transcript in FL-HCC patients. This demonstrated the utility of smaller exon-based analysis in identifying differences in transcript structure which would not be detected in larger gene or transcript-based analysis alone.

### Probabilistic & non-parametric methods

Non-parametric or probabilistic techniques such as MAJIQ, SUPPA, WHIPPET and rMATS frequently utilize Bayesian inference and/or probabilistic methodologies. ^29, 42– 44^ By avoiding assumptions about the data’s underlying distribution, these methods enable more sophisticated modelling. Consequently, in contrast to the predominantly standardized parametric exon/transcript-based techniques, event-based methods often showcase a broader array of statistical approaches ( Figure 1). A few common features can be identified, however. Often the targets for event annotations are not labelled in gene-transfer format such as splice sites, exon/intron junctions and splicing quantitative trait loci (QTLs) which must be calculated. This then allows the “Percent spliced in” (PSI) to be calculated per exon, representing the ratio of the number of transcripts containing an alternative exon versus the total number of transcripts per any given splice site. By comparing PSI values, different splicing events can then be identified and explored through splice graphs and sashimi plots. An example of non-parametric tool usage was in the mapping of splicing events in the rice (Oryza sativa) transcriptome, revealing prevalent AS under deprived nutrient conditions. ^45^ Importantly, this study utilised rMATs to reveal the underlying exon-intron structure of key nutrient transporter genes.

Some tools possess features for specific utility in certain scenarios. NOISeq is a non-parametric differential expression tool that is specifically designed to handle smaller numbers of biological replicates through its noise model. ^46^ For more complex modelling, tools such as GLiMMPs (Generalized Linear Mixed Model for Pedigree Data with Population Substructure) employ mixed-effects models to account for both fixed and random effects such as genetic family substructure. ^47^ Beyond splicing, the modular tool IsoformSwitchAnalyzeR facilitates analysis on spliced transcript quality such as Nonsense Mediated Decay (NMD) sensitivity, Intrinsically Disordered Regions (IDR) and protein domains. ^39^ Increasingly, deep learning-based approaches are being utilised to improve the accuracy of differential splicing predictions leveraging publicly available RNASeq data such as with DARTs and Bisbee. ^48, 49^

## Popularity & developer maintenance of methods

**Figure 2.**
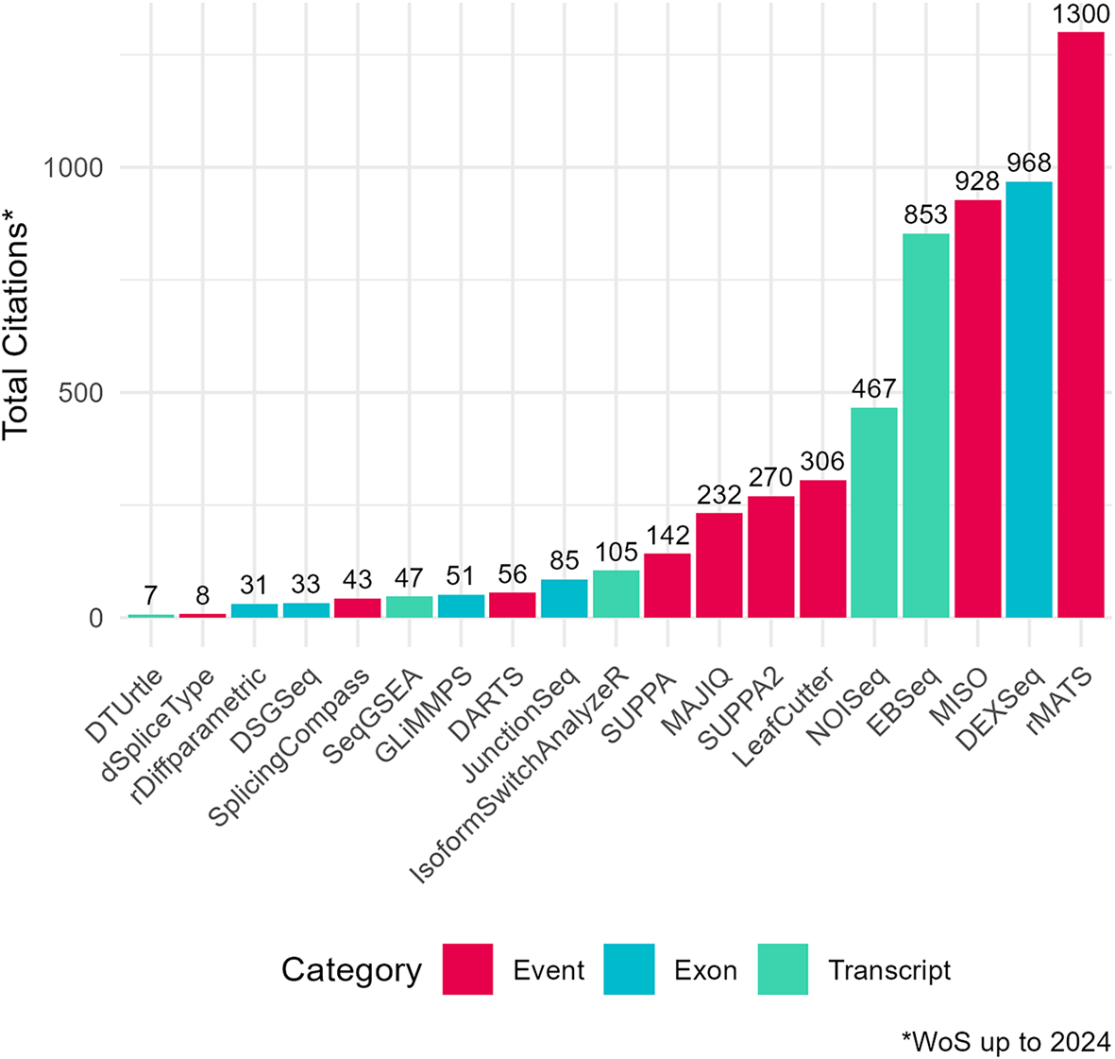
Citation counts of differential splicing tools (2010–2024) from Web of Science (WoS) Data. Total citation counts for surveyed differential splicing tools (2010–2024) from the Web of Science Data Portal (WoS). Tools are categorized by analysis level: event, exon, or transcript. DRIMSeq’s original paper was excluded from the citation frequency analysis as it was not indexed in WoS. Certain data included herein are derived from Clarivate Web of Science. © Copyright Clarivate 2023. All rights reserved.Total citation counts for surveyed differential splicing tools (2010–2024) from the Web of Science Data Portal (WoS). Tools are categorized by analysis level: event, exon, or transcript. DRIMSeq’s original paper was excluded from the citation frequency analysis as it was not indexed in WoS. Certain data included herein are derived from Clarivate Web of Science. © Copyright Clarivate 2023. All rights reserved.

**Figure 3.**
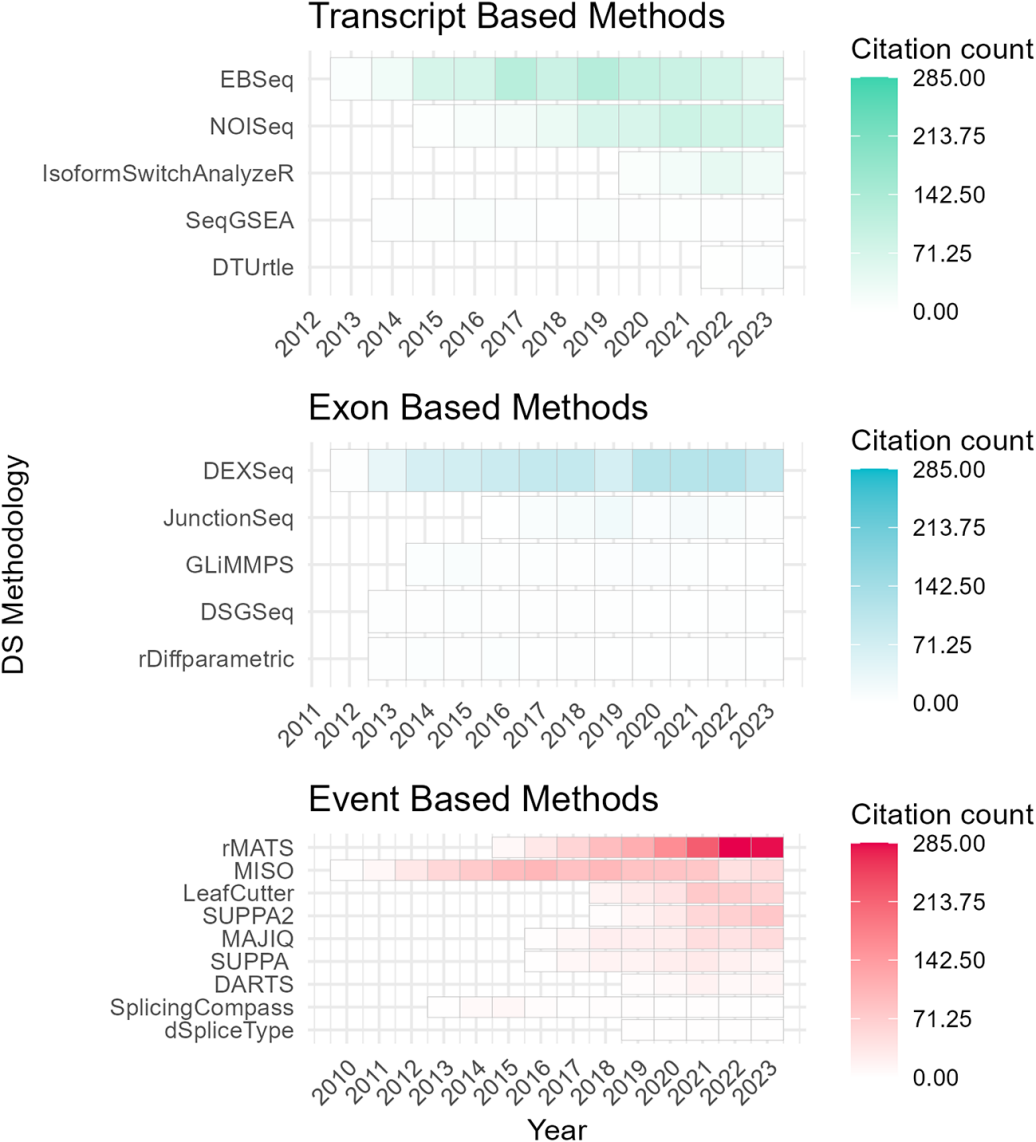
Citation trends of differential splicing tools (2010–2024) from Web of Science (WoS) Data. Annual citation frequency for current differential splicing tools (2010–2024) from Web of Science (WoS). Tools are categorized by analysis level: event, exon, or transcript. DRIMSeq’s original paper is excluded as it is not indexed in WoS. Certain data included herein are derived from Clarivate Web of Science. © Copyright Clarivate 2023. All rights reserved.

**Figure 4.**
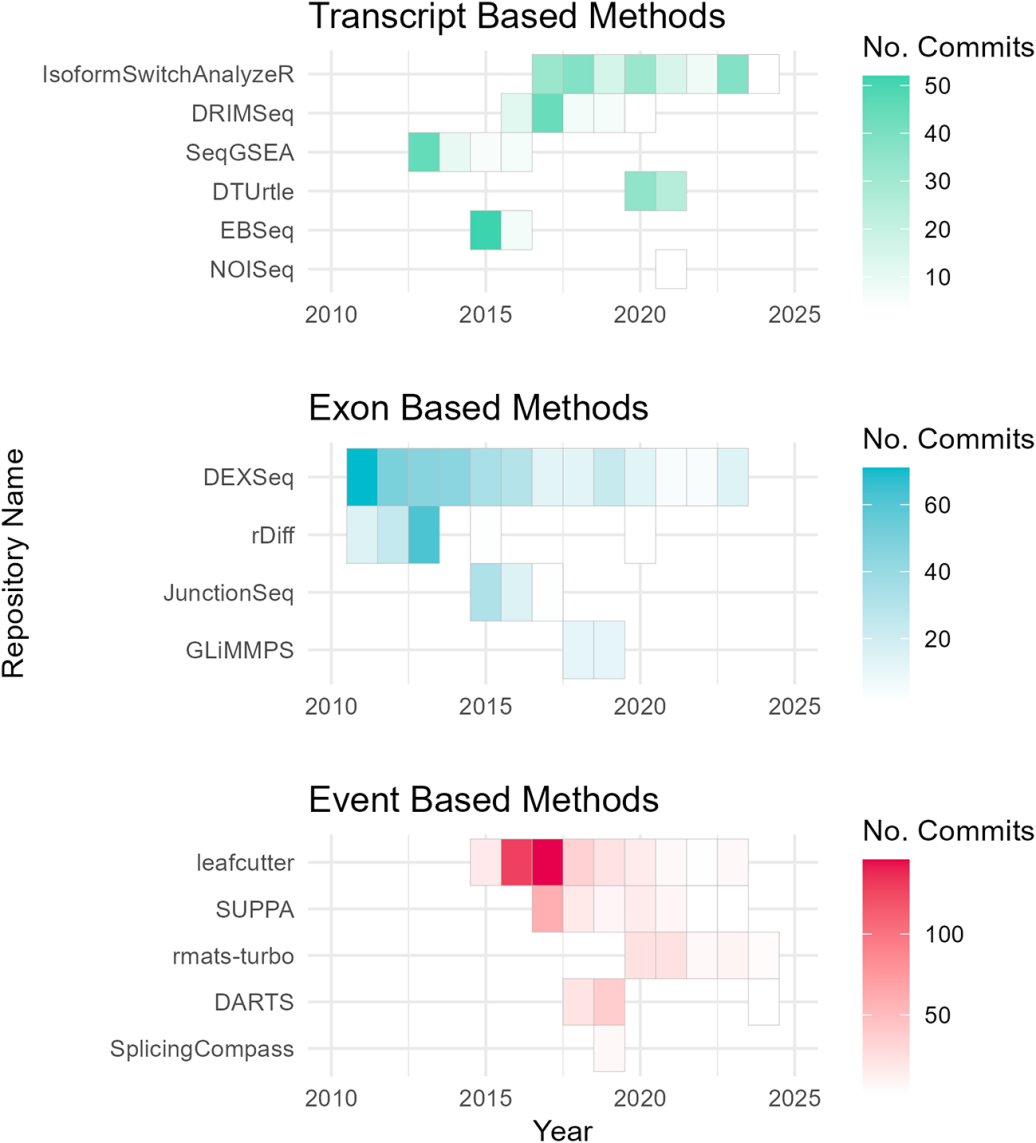
Developer maintenance of differential splicing tools. Annual GitHub repository commits (2010–2024) by category, highlighting community-led maintenance of differential splicing tools. Tools without GitHub pages (MAJIQ, MISO, DSGseq, and dSpliceType) were excluded from the analysis.

To assess the academic popularity of tools, a citation and developer engagement analysis of original research articles within the Web of Science (WoS) domain and the respective GitHub website domains (if applicable). The assessment spanned from 2010 to 2024 and encompassed 19 original papers on various differential splicing tools. Notably, the citation counts for these splicing tools were considerably lower compared to conventional RNA-Seq differential expression analysis tools. For instance, while the general purpose DGE/DTE tool DESeq2 amassed a total of 35,887 citations during the same period, citations for differential splicing tools ranged from 7 to 1300 ( Figure 2). This discrepancy may pose challenges for researchers seeking resources and workflows specific to differential splicing analysis. Additionally, the importance of developer support cannot be understated, as it directly influences the usability and longevity of software tools. Notably, differential splicing tools such as DEXSeq, EBSeq, rMATS, SUPPA2, and MAJIQ ^29, 42, 43, 50, 51^ have shown increasing usage and ongoing developer engagement, as evidenced by their growing citation counts and sustained support ( Figure 3; Figure 4). One possible explanation for the lower citation rates observed in exon/transcript-based methodologies could be the broader adoption of general-purpose differential expression workflows, like DESeq2 that can employ DTE. ^23^ Researchers may prefer more explicit splicing event-based tools for targeted splicing analyses and defer to DTE for transcript-based analyses. While the nuances between DTU and DTE may not be a primary focus for many researchers, it is a distinction worth noting in the context of differential splicing analysis.

The decision between exon/transcript-level (typically parametric) and event-level (typically non-parametric) analyses hinges on several factors, including the particular scientific inquiry, data accessibility, and the level of granularity required to address the research goal. In certain scenarios, integrating both methodologies could offer a more holistic understanding of splicing control mechanisms and their biological significance.

## Benchmarking of methods is difficult

To evaluate the quality of differential splicing bioinformatics tools, several benchmarks have been conducted to date. Benchmarking either the accuracy or the computational performance of methods can be challenging due to several factors. A primary obstacle is the lack of ground truth splicing quantifications. Often, benchmarks rely on small sets of experimentally validated splicing events as a reference. For instance, a 2019 systematic evaluation of 10 differential splicing tools tested 62 qPCR-validated differentially spliced genes across four human and mouse cancer datasets (breast, lung, prostate, and mouse lung). ^52^ This study found that rMATS and SUPPA2 exhibited higher sensitivity and precision in datasets with large library sizes, high sequencing depth, and low inter-replicate variability. While MAJIQ excelled at detecting complex splicing events (e.g., multiple exon skipping) but required greater memory and run time. Performance variability was attributed to RNA-seq data characteristics, including library size (total sequenced reads), sequencing depth (average coverage per nucleotide), positional bias (e.g., 3′ bias from poly-A selection), and the quality of reference annotations for the organism under study.

To mitigate these issues, some papers use simulated data to explore the impact of varying replicate numbers, sequencing depth, and inter-replicate variability within the data. ^53– 55^ For example, a benchmark using RSEM-based simulated data derived from a human prostate cancer dataset (GSE22260 ^56^) found that workflows based on DESeq2 and Limma outperformed others in accuracy for high-depth data with multiple replicates, while NOISeq maintained robustness across variable library sizes. ^54^ Another comparison utilised a combination of experimental and simulated Arabidopsis heat shock RNASeq datasets using the Flux Simulator tool. ^57^ However, simulated data often fails to capture the complexity of biological data, including outliers and technical biases, limiting its generalizability.

The consensus drawn from these three benchmarks, indicate that tool performance varies significantly based on data quality and analysis goals. ^52^^,^^54^ For datasets with large library sizes, high sequencing depth, and low variability, DESeq2/DEXSeq, Limma, and rMATS excel in accuracy and speed, with Limma and NOISeq requiring lower memory and run times, making them ideal for large-scale analyses. Conversely, MAJIQ is better suited for complex splicing patterns, including those potentially involving TSS and APA, despite higher computational demands. Developer updates improve tool functionality, making benchmark results time-sensitive as newer versions may outperform older ones. Community-led maintenance efforts therefore, consistently enhance the functionality and reliability of tools over time. Rather than aiming for a singular optimal tool for differential splicing analysis, researchers should contemplate employing a suite of tools tailored to address specific inquiries.

## Method recommendations

**Figure 5.**
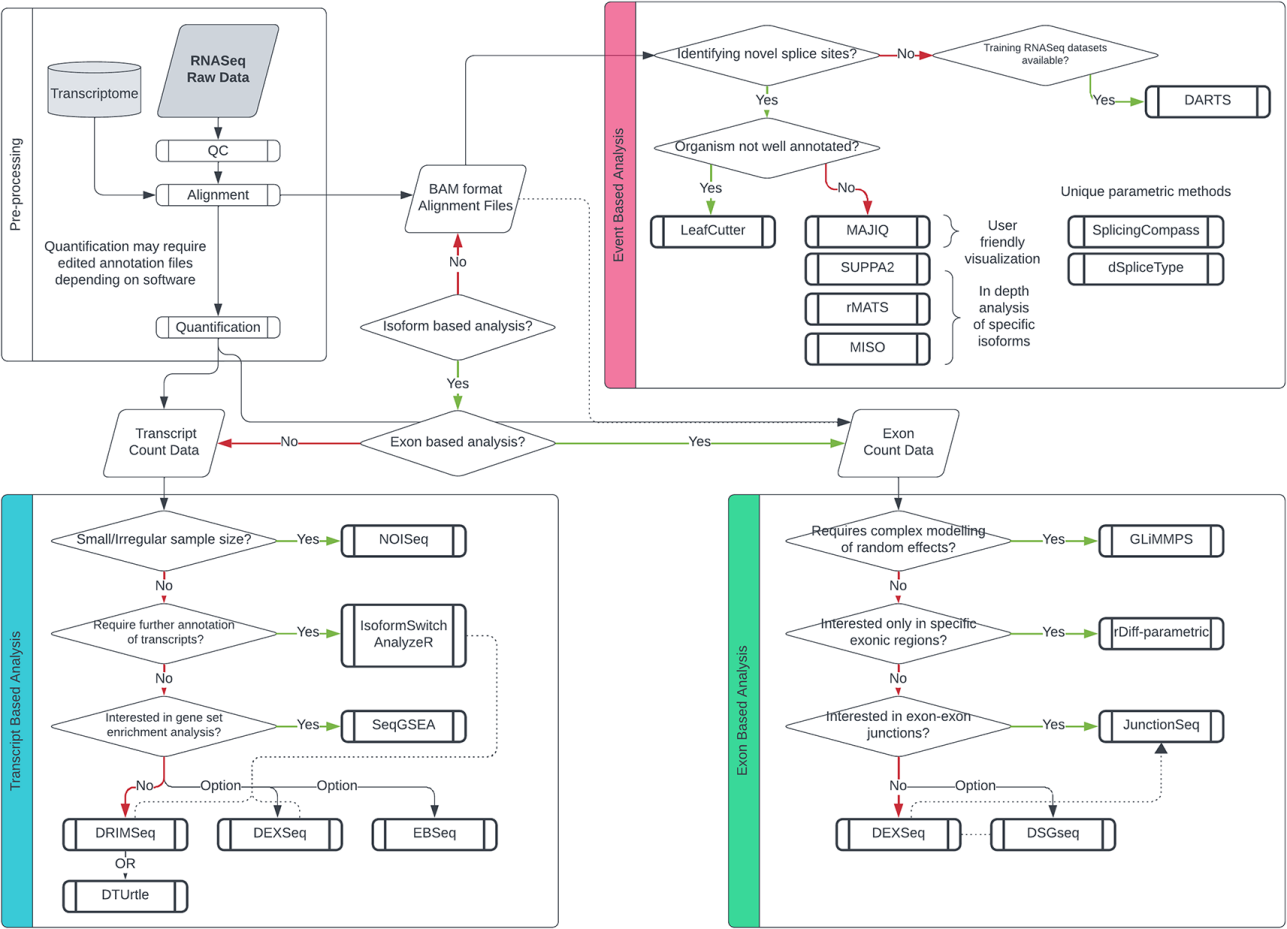
Guideline for differential splicing tool selection based on experimental parameters. Decision tree for differential splicing analysis, categorized by three branches based on the level of analysis. Transcript-based methods are represented in blue, exon-based methods in pink, and event-based methods in yellow.

A diagram outlining optimal tool selection is provided to guide prospective AS researchers ( Figure 5). Researchers should first evaluate the scope and objectives of their analysis. For detecting global, transcriptome-wide changes in transcript usage, transcript-based tools like DEXSeq or DRIMSeq are recommended for differential transcript usage (DTU) analysis, as they model transcript-level counts across the entire transcriptome, leveraging high-quality annotations to identify global shifts in isoform expression. ^40^ However, when focusing on specific transcripts or splicing events, exon- and event-based tools like rMATS, SUPPA2, or MISO provide greater detail, accurately quantifying exon inclusion or specific splicing events (e.g., exon skipping, alternative splice sites) in datasets with large library sizes and high sequencing depth. Nonetheless, variations in experimental parameters such as sample size or covariate inclusion may necessitate alternative approaches.

If the objective is to uncover novel transcripts, an exon-based parametric approach might be better suited. This choice circumvents the challenges associated with isoform deconvolution and the breadth of transcript annotation, given the smaller exonic regions. For general-purpose differential exon usage (DEU) analysis, DEXSeq is widely used due to its robust statistical framework and active maintenance within Bioconductor, while rMATS is highly cited for its accuracy and speed in exon usage quantification, making both complementary choices for detailed exon-level analysis. ^23^^,^^43^ However, again intricacies within the data may prompt the usage of more specialised alternatives. Transcript- and exon-based methods support top-down visualizations like MA/Volcano plots, heatmaps, and proportional transcript/exon graphs, suitable for summarizing global or exon-specific changes. If the analysis aims to visualise the movement of exons/introns and splice sites, then an event-based protocol would be more appropriate. Generally, tools such as rMATs, SUPPA2 and MISO offer comprehensive and detailed splicing event analysis. ^42, 43, 58^

Commonly, sashimi plots are the best method to visualise splice junctions from aligned data with events annotated, although this can also be plotted separately in IGV. ^59^ For user-friendly visualization, MAJIQ offers a summative HTML-based visualizer for complex events such as exitrons or orphan junctions. ^29^ Usability varies by tool interface: DEXSeq and DRIMSeq integrate with R environments, while rMATS, SUPPA2, and MAJIQ use command-line interfaces, runnable in IDEs like Visual Studio Code, with MAJIQ and SUPPA2 offering graphical outputs for broader accessibility. For organisms with poor annotation quality, annotation-free methods like LeafCutter are valuable alternatives. ^60, 61^ Optional steps, such as using Portcullis to filter false splice junctions, can enhance data quality by addressing misalignments common in short-read data. ^62^ For most analyses, DEU or DTU approaches (e.g., DEXSeq, rMATS) are recommended for their interpretability and robustness, with DTU tools preferred for transcriptome-wide insights and event-based tools for detailed, transcript-specific splicing analysis.

## Discussion

While the repertoire of tools for differential splicing (DS) analysis has expanded over the past two decades, their effectiveness remains tied to RNASeq technology capabilities. Since 2010, long-read RNAseq, enabled by technologies like Oxford Nanopore Technologies (ONT) and PacBio’s single-molecule real-time (SMRT), has offered read lengths of 10kb to 100kb, with ultra-long reads reaching 1-2 Mb. ^63– 65^ This allows reconstruction of full-length transcript isoforms in a single read, bypassing deconvolution issues from multiple mapping and improving detection of known transcripts, novel splice variants, and fusion genes. However, long-read sequencing remains costly, often requiring hybridization with short-read RNAseq to achieve high accuracy (up to 99.5%). ^66^ Tools like StringTie2, exemplify this hybrid approach, combining short- and long-read data to enhance transcript assembly accuracy by leveraging the precision of short reads and the isoform resolution of long reads. ^67^ Consequently, short-read-based DS tools, such as IsoformSwitchAnalyzeR’s DEXSeq-based DTU workflow, remain highly relevant, as demonstrated by their successful application to ONT long-read data. ^23, 39, 68^

The high cost of long-read RNAseq underscores the continued importance of short-read DS tools, especially given the wealth of publicly available short-read RNAseq datasets in repositories like NCBI Gene Expression Omnibus (GEO), ^69^ EMBL-EBI ArrayExpress, ^70^ and Sequence Read Archive (SRA). ^71^ These thousands of datasets enable meta-analyses that yield novel biological insights without the expense of new sequencing. As interest in alternative splicing grows, advances in statistical methods and sequencing technologies are overcoming technical limitations, improving transcript alignment and simplifying computational workflows. Streamlined and modular workflows, such as those provided by Nextflow and the nf-core/rnasplice pipeline, empower researchers to create tailored AS pipelines with minimal setup effort, leveraging containerization to bypass installation challenges. ^72^ The synergy of cost-effective short-read tools, hybrid strategies, and extensive public datasets ensures a promising future for alternative splicing analysis, deepening our understanding of transcriptomic regulation and its functional significance.

## Ethical approval and consent statement

Ethical approval and consent were not required.

## Data Availability

No data associated with this article. Zenodo: Selecting differential splicing methods: Practical considerations https://doi.org/10.5281/zenodo.14293573. ^73^ The repository contains the following underlying data:
• Supplementary Table 1.docx: Statistical details on differential splicing tools. • citations_2023.csv: WoS citation count for differential splicing tools. •
citations_year_plot_new.R: R script to visualise citation trends. •
github_repos_txt: Github repository locations cloned on 20.02.2024. • github_repos.R: Github maintenance analysis and visualisation. •
citations_2023.xlsx

## Software availability statement

• https://github.com/bjdraper/Selecting-differential-splicing-methods-Practical-Considerations---R-Scripts-and-Data
• Data are available under the terms of the Creative Commons Attribution 4.0 International license (CC-BY 1.0).

Archived software available from: 10.5281/zenodo.14293573

github_repos.R

citations_year_plot_new.R

Data are available under the terms of the Creative Commons Attribution 4.0 International license (CC-BY 4.0).
