# Supplementary material for: Selecting differential splicing methods: Practical considerations for short-read RNA sequencing
Source: F1000Res. 2025 May 30;14:47. Originally published 2025 Jan 8. [Version 2] doi: 10.12688/f1000research.155223.2 (PMC12308171; doi:10.12688/f1000research.155223.2)
Supplement: Peer review history

# Reviewer report: Reviewer response for version 2

**DOI:** 10.5256/f1000research.182560.r389377

**Published:** 2025-08-28

**Authors:** Juan Valcarcel, Monica Salinas

**Affiliations:** 1 Genome Biology, Centre de Regulacio Genomica (Ringgold ID: 16372), Barcelona, Catalonia, Spain; 2 The Barcelona Institute of Science and Technology, Barcelona, Spain

This review by Draper et al. provides a comprehensive and well-curated overview of the available tools for RNA-Seq-based alternative splicing (AS) analysis. While differential gene expression remains the most common application of RNA-Seq data, the authors successfully bring attention to the expanding repertoire of computational methods dedicated to AS. The manuscript is clearly written, well-structured, and the figures are informative; in particular the decision-tree diagram in Figure 5, which serves as a helpful visual guide for tool selection.

 Requested revisions:

 1) It is not entirely clear whether the distinction between exon-based and event-based approaches refers solely to whether different types of splicing events (e.g., retained introns, alternative splice sites, etc.)or only exons are considered; or if there is an additional level of distinction. A more explicit definition of this stratification would help improve clarity and guide readers in understanding the differences between both strategies.

 2)Vast-tools, a widely used tool in the AS community, is notably absent from the current manuscript. The main publication describing Vast-tools has garnered >400 citations and the tool is actively maintained with comprehensive documentation available on GitHub. It meets the inclusion criteria set by the authors (ie citation impact and repository maintenance). Vast-tools enables PSI-based quantification of >700000 annotated splicing events and is part of a broader analytical framework that includes matt (for downstream analysis of AS event features) and vastDB (a comprehensive database of AS profiles). Including this tool in the review would provide a more complete representation of the field and would be useful for readers.

 Associated references:

  

- Vast-tools: https://doi.org/10.1101/gr.220962.117 (Ref 1)

 

- Vast-tools GitHub: https://github.com/vastgroup/vast-tools

 

- Matt: https://doi.org/10.1093/bioinformatics/bty606 (Ref 2)

 

- Matt GitHub: https://gitlab.com/aghr/matt

 

- VastDB: https://vastdb.crg.eu/wiki/Main_Page

3) Sequencing depth is a critical parameter that is often underappreciated in splicing analyses. The standard 30M reads per sample, typically sufficient for gene expression studies, are generally inadequate for reliable quantification of splicing events—particularly for low-abundance isoforms. This limitation should be emphasized more clearly in the manuscript, especially in the context of data generation and when selecting publicly available datasets. Many public RNA-Seq datasets were not originally designed for AS analysis, which can severely limit downstream interpretations when re-analysed.

 4) About Tools Comparison:

 4.1) While the statistical frameworks underlying each tool are briefly mentioned, a more detailed explanation of their analytical approaches (beyond simply naming the statistical test used) would enhance the review’s educational value, especially for non-expert readers.

 4.2) The manuscript could benefit from a more explicit discussion on whether each tool relies on predefined annotations or performs de novo detection of splice junctions. This distinction is crucial depending on the biological question (e.g., whether the aim is to identify novel or neojunctions, or to compare known events across conditions).

 4.3) Similarly, an overview comparing the output metrics used by the different tools would be highly informative and would help readers determine which software best suits their analysis goals. In those cases that do not rely on PSI values, which alternative metrics are used?

 5) On page 5, when comparing expression normalization metrics such as CPM, TPM, and RPKM, a brief explanation of the contexts in which each metric is most appropriate would be helpful.

 6)In the discussion on long-read sequencing technologies, it would be useful to indicate which of the reviewed tools are compatible with long-read RNA-Seq data, and which are restricted to short-read inputs. This information could, for example, be conveyed as an additional annotation in one of the existing figures (e.g., Figure 5), rather than in the main text.

Is the review written in accessible language?

Yes

Are all factual statements correct and adequately supported by citations?

Yes

Are the conclusions drawn appropriate in the context of the current research literature?

Yes

Is the topic of the review discussed comprehensively in the context of the current literature?

Yes

Reviewer Expertise:

Alternative splicing, RNA biology

We confirm that we have read this submission and believe that we have an appropriate level of expertise to confirm that it is of an acceptable scientific standard, however we have significant reservations, as outlined above.

## References

[1] : An atlas of alternative splicing profiles and functional associations reveals new regulatory programs and genes that simultaneously express multiple major isoforms. *Genome Research* .2017;27(10) : 10.1101/gr.220962.117 1759-1768 10.1101/gr.220962.117 PMC563003928855263

[2] : Matt : Unix tools for alternative splicing analysis. *Bioinformatics* .2019;35(1) : 10.1093/bioinformatics/bty606 130-132 10.1093/bioinformatics/bty606 30010778

# Reviewer report: Reviewer response for version 2

**DOI:** 10.5256/f1000research.182560.r397528

**Published:** 2025-08-13

**Authors:** Zhaoqi Liu

**Affiliations:** 1 China National Center for Bioinformation, Beijing, China

**Summary:**

 This manuscript provides a comprehensive review of differential alternative splicing analysis methods primarily based on short-read RNA sequencing data. The authors cover a wide range of computational tools and statistical frameworks, including both parametric and non-parametric approaches, and discuss their applications, strengths, and limitations. Overall, the article offers a valuable resource for researchers aiming to select appropriate tools for AS analysis and to understand the landscape of current methodologies.

**Minor Comments:**

1. **User Experience and Usability: **It would strengthen the review to include a systematic comparison of installation complexity, dependency requirements, and user-friendliness across the reviewed tools. Furthermore, a discussion on each tool’s visualization capabilities—such as graphical user interfaces, scripting options—would greatly benefit non-expert users and enhance the practical guidance of the review.
2. **Computational Resource Requirements: **Given that large-scale RNA-Seq datasets are increasingly common, the authors are encouraged to comment on the computational efficiency of the tools, including CPU and memory demands, support for multi-threading or distributed computing. This would provide readers with a better understanding of which tools are most suitable for extensive datasets.
3. **Future Directions: **Besides short-read and long-read sequencing integration, more elaborate coverage of emerging trends would be valuable. Specifically, a deeper analysis of deep learning techniques for splicing event detection and interpretation should be considered. Additionally, current challenges and prospects in single-cell splicing analysis could be addressed in more detail, especially how short-read tools are applied in single-cell contexts by aggregating data into pseudo-bulk samples.

**Conclusion:**

 This manuscript is well-organized and informative. Addressing the above points would further enhance its clarity, practical utility, and relevance to current and future research trends in alternative splicing analysis.

Is the review written in accessible language?

Yes

Are all factual statements correct and adequately supported by citations?

Yes

Are the conclusions drawn appropriate in the context of the current research literature?

Yes

Is the topic of the review discussed comprehensively in the context of the current literature?

Yes

Reviewer Expertise:

Computational cancer genomics with a focus on RNA splicing dysregulation in human cancer

I confirm that I have read this submission and believe that I have an appropriate level of expertise to confirm that it is of an acceptable scientific standard.

# Reviewer report: Reviewer response for version 2

**DOI:** 10.5256/f1000research.182560.r397523

**Published:** 2025-07-29

**Authors:** Julia Olivieri

**Affiliations:** 1 University of the Pacific, California, USA

This paper provides a review of alternative splicing (AS) analysis software for short-read RNA sequencing data. Tool popularity and active maintenance are compared using metrics such as citation count and number of GitHub commits. The flowchart provided in Figure 5 is especially helpful for researchers determining which approach is best for their use case. This review is written in accessible language, and breaks the different approaches down into helpful categories, such as parametric vs nonparametric and differential transcript expression (DTE) vs differential exon usage (DEU) vs differential splicing events (DSE). 

 This paper will be a useful resource as alternative splicing analysis on short-read RNA sequencing data becomes more prevalent. However, I have several concerns with the paper that I recommend addressing.

**Concerns that should be addressed to make the article scientifically sound:**

- The abstract states that 22 tools are analyzed. However, the tools are not fully consistent throughout. DESeq2 is mentioned in the supplemental table, although it is not specifically optimized for AS analysis, and similar tools such as LIMMA and edgeR are not included in the table. Also, WHIPPET is included in the table and mentioned in the text but not included in any of the figures, and Cuffdiff2 is mentioned in the table and Figure 1, but not elsewhere. Keeping the tool list consistent would aid the clarity of the paper.
- DRIMSeq and DTUrtle are categorized as parametric models, but the text says that they use non-parametric models. This should be clarified.
- Bisbee is stated to be a deep-learning method, but this is not supported by the citation.

**Additional recommendations:**

- In Figure 5, the “isoform-based analysis” diamond breaks the flow of the chart: one cannot trace a path from the top left to this diamond. I recommend re-positioning it to fit into the flow chart more naturally. Also, I recommend adding arrows to SplicingCompass and dSpliceType in the flowchart based on when they should be used (unless they would never be recommended).
- Understanding the difference between DGE/DTE, DTU/DEU, and DSE is critical to fully understanding the paper. A figure to go along with the in-text description would be very helpful to clarify this for the reader.
- Visualization tools are mentioned several times in the manuscript. It would be helpful to include example plots, at least an example sashimi plot, because many readers are likely unfamiliar with these plot types.
- The text states about nonparametric models that “By avoiding assumptions about the data’s underlying distribution, these methods enable more sophisticated modeling.” Rather than “enable,” I would say “require.” 
- It is stated that the lower citation counts of software for AS analysis compared to DGE analysis “may pose challenges for researchers seeking resources and workflows specific to differential splicing analysis.” I am not sure why fewer citations would pose challenges - I would suggest clarifying or changing the wording (I would understand if fewer options posed challenges, or if lower usage meant less developer support).
- There are several typos, words missing, and grammatical errors that could be fixed with another round of proofreading. For example, in the supplemental table “riff-parametric” should be “rdiff-parametric” and “To assess the academic popularity of tools, a citation and developer engagement analysis of original research articles within the Web of Science (WoS) domain and the respective GitHub website domains (if applicable)” is a sentence fragment.
- The section about long-read sequencing in the discussion feels somewhat out of place based on the short-read focus of the rest of the paper. I recommend shortening this section.

Is the review written in accessible language?

Yes

Are all factual statements correct and adequately supported by citations?

Partly

Are the conclusions drawn appropriate in the context of the current research literature?

Yes

Is the topic of the review discussed comprehensively in the context of the current literature?

Yes

Reviewer Expertise:

Alternative splicing analysis, bioinformatics, biostatistics, single cell RNA sequencing

I confirm that I have read this submission and believe that I have an appropriate level of expertise to confirm that it is of an acceptable scientific standard, however I have significant reservations, as outlined above.

# Reviewer report: Reviewer response for version 2

**DOI:** 10.5256/f1000research.182560.r388756

**Published:** 2025-06-27

**Authors:** Charlotte Capitanchik

**Affiliations:** 1 UK DRI at King’s College London, London, UK

I find the updated article much improved.

Is the review written in accessible language?

Partly

Are all factual statements correct and adequately supported by citations?

Partly

Are the conclusions drawn appropriate in the context of the current research literature?

Partly

Is the topic of the review discussed comprehensively in the context of the current literature?

Yes

Reviewer Expertise:

bioinformatics, splicing, RNA biology

I confirm that I have read this submission and believe that I have an appropriate level of expertise to confirm that it is of an acceptable scientific standard.

# Reviewer report: Reviewer response for version 1

**DOI:** 10.5256/f1000research.170367.r375800

**Published:** 2025-04-25

**Authors:** Charlotte Capitanchik

**Affiliations:** 1 UK DRI at King’s College London, London, UK

The article by Draper and colleagues presents a well-researched and well-organised overview of differential splicing analysis tools for short-read RNA sequencing, which will be valuable for many researchers. The practical focus is excellent, I especially like the section on developer maintenance of tooling, which is often neglected in software review articles. The decision tree in Figure 5 is also a great framework for navigating this crowded space and I’m sure will be useful to many. 

 Despite this, in many places the use of language is imprecise: words are missing or used incorrectly, sentences are too vague—perhaps from being over-simplified in an attempt to make the text more understandable. I also find some of the recommendations are not adequately supported by evidence. Please find some comments that I hope will be helpful, below: 

**Major comments**

- The method benchmarking section needs some attention. ‘Scientific accuracy’ should be replaced by just ‘accuracy’. ‘Computational power’ should be specified, presumably you mean performance characteristics such as memory usage and run time. “Lack of ground truth to set as a reference to compare to..” is unnecessary, just say a lack of ground truth splicing quantifications. Library size and sequencing depth are the same thing? Library size and positional bias are qualities of the RNA-seq data itself, not the bioinformatic pre-processing steps. Annotation quality is not a ‘pre-processing’ step - this is a feature of the organism under study. “Achieve finer control over the ground truth” could be rephrased to something more meaningful like ‘to explore the impact of increasing variability between replicates, changing replicate numbers, sequencing depth’…..etc. “The consensus drawn from these three benchmarks is that the performance of differential splicing tools exhibits considerable variability depending on the outlined factors.” This seems weak, perhaps a more nuanced conclusion can be drawn - when data is very good, deep sequencing, low variability, which tool performs best? Which tools have the lowest run times, compute requirements .etc. I don’t think it’s sufficient to simply say its complicated - please dive more into the details here. “The ongoing evolution and upkeep of tools by developers introduce a time-dependent aspect to benchmarking. Community-led maintenance efforts consistently enhance the functionality and reliability of tools over time.” I’m not sure what this means, please clarify. 
- The discussion brings in developments in long read sequencing and is quite nice - I would suggest making this a section of its own and expanding on what is already written. Alternatively, I would consider cutting it back a bit and changing the title of the article to reflect a focus on short-read sequencing data. Perhaps one point for the discussion is that whilst new methods are developing, there remains hundreds of thousands of publicly available short read RNA sequencing datasets through which novel biological insights can still be made.
- In the section of recommendations - “For instance, if the aim is to identify known transcripts, it is advisable to opt for a parametric transcript-based tool like DEXSeq or DRIMSeq and execute a DTU study following Michael Love’s protocol.37” The citation does not support the statement - why shouldn’t researchers opt for an exon-based approach when the transcriptomic annotations are good? Also in this section, DEXSeq is recommended for DEU analysis, when rMATs is the most highly cited splicing tool and provides accurate quantifications of exon usage. “Overall, event-based methods are more suited to advanced programmers owing to their use of command-line tools over interpreters that use IDEs (Integrated development environments). For most analyses, however, a DEU or DTU-based analysis is recommended for simple interpretability and robustness.” I don’t understand, how is DEX-Seq easier to use than rMATs (for example), or MAJIQ which has extensive graphical reporting? Are you saying this because DEX-Seq is an R package so you can use RStudio?  - this doesn’t seem like a particularly helpful argument - I can run rMATs or MAJIQ or any of these using a bash script in Visual Studio Code which is also an IDE…

**Minor comments**

- Alternative splicing (AS) abbreviation is given several times throughout text and sometimes used, sometimes not - please be consistent.
- “The proposed advantage of this approach is in the smaller exonic regions rather than full isoform deconvolution.” Rephrase for clarity, presumably you mean by focusing on regions unique to distinct isoforms the tool avoids the issue of assigning ambiguous reads to isoforms.
- "A few newer methods such as DRIMSeq and DTUrtle have progressed onto non-parametric or mixed Dirichlet Multinomial Models (DMM)" ‘progression’ suggests there is some kind of hierarchy, you can just say that these models ‘use’ other distributions 
- “More complex regulatory events involve genomic features beyond exons and introns, such as alternative promoter and polyadenylation sites, which result in varying mRNA 5′ and 3′ UTR ends. However, these events are seldom included in most bioinformatics analyses, tools such as CAGER (Cap Analysis of Gene Expression) and DaPars (Dynamic Analysis of Alternative PolyAdenylation from RNA-Seq) are available for niche research” I wouldn’t say these events are more complex from a biological standpoint. The issue in analysis of alternative TSS use and APA is that short read sequencing with typical library preparation methods (e.g. random hexamer priming) won’t have good coverage of exact transcript 5’ and 3’ ends. Therefore typically library preparations with mRNA cap capture (CAGE) or 3’ end sequencing (eg. Quantseq) are used when this is the analysis goal. Also, to be a pedant, 3’UTRs and 5’UTRs are exons.
- The discussion mentions Nextflow, and nf-core is mentioned earlier, but it might be nice to specifically mention the efforts of nf-core/rnasplice. As you know, one of the benefits of these pipelines is that everything is containerised so you don’t have to mess about installing everything. Generally speaking, one of the barriers to using tools can be installation - of the presented tools there is quite a range of levels of developer investment in making the tools easy to install. Some are in bioconda or bioconductor and have containers, others you have to contact the authors to get permission to download (MAJIQ!) - this might be related to the amount of citations that tools get. It would be nice (but not necessary) to address this too.

Is the review written in accessible language?

Partly

Are all factual statements correct and adequately supported by citations?

Partly

Are the conclusions drawn appropriate in the context of the current research literature?

Partly

Is the topic of the review discussed comprehensively in the context of the current literature?

Yes

Reviewer Expertise:

bioinformatics, splicing, RNA biology

I confirm that I have read this submission and believe that I have an appropriate level of expertise to confirm that it is of an acceptable scientific standard, however I have significant reservations, as outlined above.

# Response

**Published:** 2025-05-21

**Authors:** Ben Draper

**Affiliations:** The University of Sheffield Department of Chemical and Biological Engineering, Sheffield, England, UK

Dear Dr. Capitanchik,

 Thank you for your thorough and insightful review of our manuscript. Your comments on the benchmarking, recommendations, long-read discussion, and minor edits have significantly improved the manuscript’s clarity and accuracy. We have incorporated minimal changes to address your concerns, ensuring the revisions align with your suggestions while maintaining the manuscript’s focus.

 For the benchmarking section, we corrected terminology (“accuracy” instead of “scientific accuracy,” “computational performance” instead of “computational power”), clarified that library size, positional bias, and annotation quality are dataset characteristics, not pre-processing steps, and rephrased vague terms (e.g., “lack of ground truth” to “lack of comprehensive ground truth splicing quantifications”). We added specific examples drawn from the benchmarks (e.g., DEXSeq, rMATS, NOISEQ) to strengthen the conclusions.

 In the recommendations section, we clarified our positions on programming environments, developer and community support, making sure not to discriminate harshly against command line-based tools. We believe this is still worth mentioning, however, as in our experience, the programming platform matters for accessibility.

 In the discussion, we opted for shortening the long-read section for brevity and making the article more focused towards short-read. We have added a few minor points in agreement with Dr. Donega. We agree with all the minor edits (e.g., consistent AS acronym usage, clarified rDiff-parametric, revised TSS/APA) that were made as requested.

 We believe these changes should address your concerns effectively.

 Best wishes

 Ben J. Draper

 University of Sheffield

# Reviewer report: Reviewer response for version 1

**DOI:** 10.5256/f1000research.170367.r375793

**Published:** 2025-04-22

**Authors:** Stefano Donega

**Affiliations:** 1 National Institute on Aging, Bethesda, USA

In this review, Dr. Draper, Dunning, and James provide a very good comprehensive and well-written summary of the tools applied to the investigation of alternative splicing, with a thorough literature perspective and a detailed guide to choosing the most appropriate statistical methods and software. While I really appreciated and enjoyed reading the manuscript, I would like to provide a few comments that I believe would enhance and elevate the quality of the work:

- In general, the entire manuscript discusses methods that directly apply to short-read platforms. Therefore, I think this should be better highlighted both in the manuscript title and throughout the whole review.
- The long-read platforms appear only in the discussion section. I recommend the authors dedicate a separate paragraph to them, independent of the discussion, while keeping the discussion to connect together the main findings investigated in the main text.

Now, I will provide some minor comments:

- In a recent Nature Aging paper, Ferrucci et al. 2022 (Ref 1) discussed the "energy-splicing axis hypothesis on aging," which is worthy of mentioning in the introductory paragraph on the importance of splicing.
- There have been efforts to clarify modern nomenclature in gene expression studies, and guidelines were recently proposed to increase precision and clarity when communicating about gene expression, most notably to reserve 'gene' for the DNA template and 'transcript' for the RNA transcribed from that gene (Cunningham ASG, et al., 2024 [Ref 2]). I suggest authors consider aligning some definitions found in the manuscript with these guidelines.
- There is no mention of the possibility of combining short- and long-read sequencing to enhance quantity and quality of results. I strongly suggest the authors include in their review a section on "StringTie" which utilizes both short and long RNA-seq reads for transcript assembly to generate a hybrid strategy (Shumate A, et al., 2022 [Ref 3]).

After these improvements, I am confident this article will be highly cited in the field.

Is the review written in accessible language?

Yes

Are all factual statements correct and adequately supported by citations?

Yes

Are the conclusions drawn appropriate in the context of the current research literature?

Partly

Is the topic of the review discussed comprehensively in the context of the current literature?

Yes

Reviewer Expertise:

Aging, muscle, mitochondria, energy, hypoxia, exercise

I confirm that I have read this submission and believe that I have an appropriate level of expertise to confirm that it is of an acceptable scientific standard, however I have significant reservations, as outlined above.

## References

[1] : The energy-splicing resilience axis hypothesis of aging. *Nat Aging* .2022;2(3) : 10.1038/s43587-022-00189-w 182-185 10.1038/s43587-022-00189-w 37118371 PMC11330567

[2] : Striving for clarity in language about gene expression. *Nucleic Acids Res* .2024;52(18) : 10.1093/nar/gkae764 10747-10753 10.1093/nar/gkae764 39271127 PMC11472038

[3] : Improved transcriptome assembly using a hybrid of long and short reads with StringTie. *PLoS Comput Biol* .2022;18(6) : 10.1371/journal.pcbi.1009730 e1009730 10.1371/journal.pcbi.1009730 35648784 PMC9191730

# Response

**Published:** 2025-05-21

**Authors:** Ben Draper

**Affiliations:** The University of Sheffield Department of Chemical and Biological Engineering, Sheffield, England, UK

Dear Dr. Donega,

 First of all, thank you for reviewing the article. I appreciate the time you took and the constructive feedback you gave me to improve this work. 

 The title was revised to “Selecting Differential Splicing Methods: Practical Considerations for Short-Read RNA Sequencing” to emphasise short-read platforms, and the abstract and introduction now explicitly state this focus. I am hesitant to expand and write a full section on long-read technology, as this isn't really my field of expertise. Therefore, we decided to streamline this section in line with Dr Capitanchik's recommendations while weaving in the hybridised approaches of current short-read technologies.

 I agree with the minor points and have addressed these by including the recommended citations in the introduction and discussion. 

 These changes align the manuscript with your recommendations, maintaining its comprehensive scope while clarifying its primary focus on short-read RNA-seq. 

 Sincerely,

 Ben J. Draper

 University of Sheffield
